# Supplementary material for: Ecological context structures duplication and mobilization of antibiotic and metal resistance genes in bacteria
Source: bioRxiv. 2026 Feb 6:2026.02.03.703612. Preprint. [Version 1] doi: 10.64898/2026.02.03.703612 (PMC12889720; doi:10.64898/2026.02.03.703612)
Supplement: Supplement 3 [file media-3.pdf]

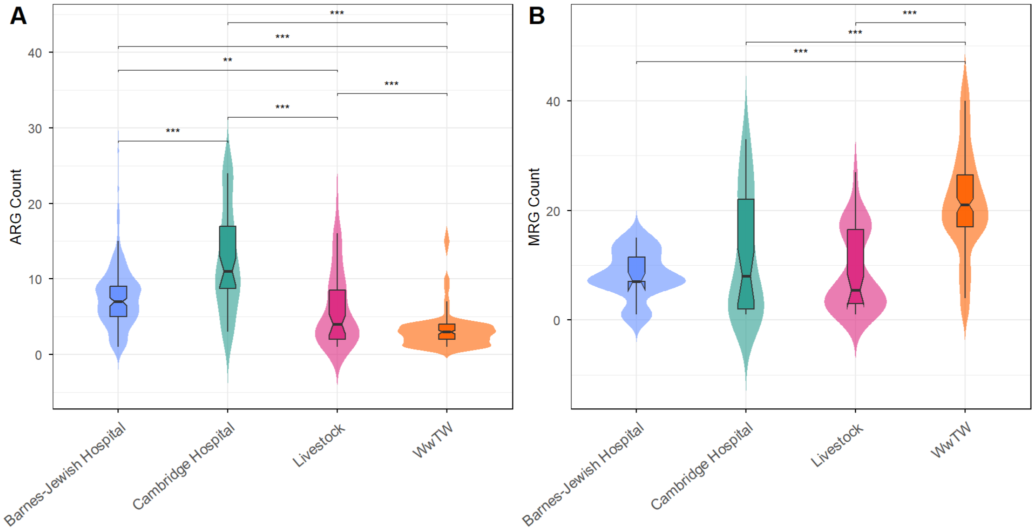

Figure S1: Abundance of ARGs (**A**) and MRGs (**B**) per genome across the four environments. Violin plots show distributions of gene counts, with embedded boxplots indicating the median and interquartile range. Analyses were performed with *E. coli* filtered from all environments except Barnes–Jewish Hospital, which consists exclusively of *E. coli* isolates and was therefore retained. $*p < 0.05$, $**p < 0.01$, $***p < 0.001$ (see *Methods*).

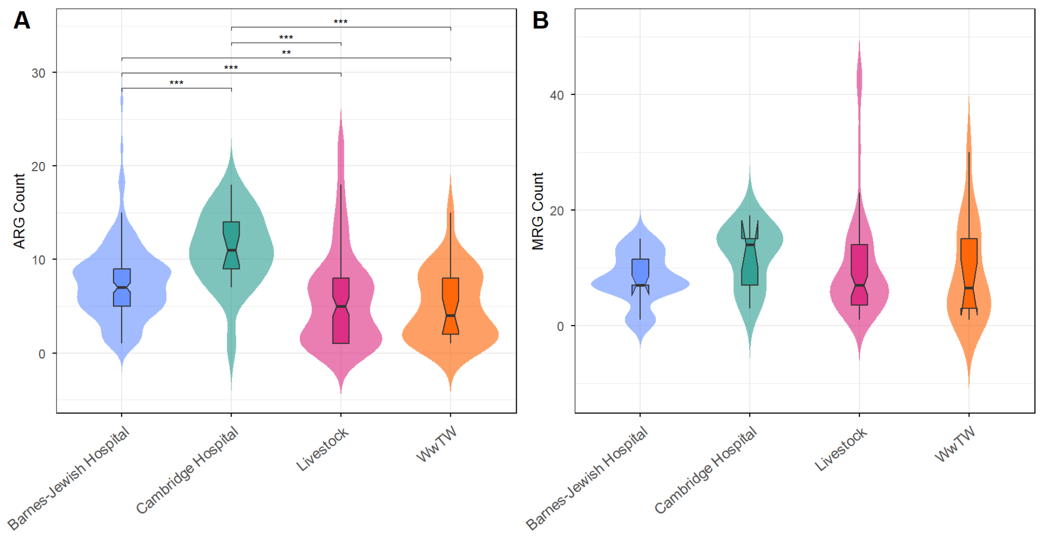

Figure S2: Abundance of ARGs (**A**) and MRGs (**B**) per genome across the four environments. Violin plots show distributions of gene counts, with embedded boxplots indicating the median and interquartile range. Analyses were performed with *E. coli*-only. *$p < 0.05$, **$p < 0.01$, ***$p < 0.001$ (see *Methods*).
